# Phytosynthesis of BiVO_4_ nanorods using *Hyphaene thebaica* for diverse biomedical applications

**DOI:** 10.1186/s13568-019-0923-1

**Published:** 2019-12-12

**Authors:** Hamza Elsayed Ahmed Mohamed, Shakeeb Afridi, Ali Talha Khalil, Tanzeel Zohra, Muhammad Masroor Alam, Aamir Ikram, Zabta Khan Shinwari, Malik Maaza

**Affiliations:** 1 UNESCO UNISA Africa Chair in Nanosciences and Nanotechnology, College of Graduate Studies, University of South Africa, Pretoria, South Africa; 2 NANOAFNET (Nanosciences African Network), Materials Research Department, iThemba LABS, Cape Town, South Africa; 3 Department of Biotechnology, Quaid-i-Azam University, Islamabad, Pakistan; 4 Department of Biotechnology, Qarshi University, Lahore, Pakistan; 5 National Institute of Health, Islamabad, Pakistan; 6 Pakistan Academy of Sciences, Islamabad, Pakistan

**Keywords:** Bismuth vanadate, BiVO_4_, Nanorods, *Hyphaene thebaica*, Antimicrobial, Antioxidant, Antiviral

## Abstract

Biosynthesis of bismuth vanadate (BiVO_4_) nanorods was performed using dried fruit extracts of *Hyphaene thebaica* as a cost effective reducing and stabilizing agent. XRD, DRS, FTIR, zeta potential, Raman, HR-SEM, HR-TEM, EDS and SAED were used to study the main physical properties while the biological properties were established by performing diverse assays. The zeta potential is reported as − 5.21 mV. FTIR indicated Bi–O and V–O vibrations at 640 cm^−1^ and 700 cm^−1^/1120 cm^−1^. Characteristic Raman modes were observed at 166 cm^−1^, 325 cm^−1^ and 787 cm^−1^. High resolution scanning and transmission electron micrographs revealed a rod like morphology of the BiVO_4_. *Bacillus subtilis*, *Klebsiella pneumonia*, *Fusarium solani* indicated highest susceptibility to the different doses of BiVO_4_ nanorods. Significant protein kinase inhibition is reported for BiVO_4_ nanorods which suggests their potential anticancer properties. The nanorods revealed good DPPH free radical scavenging potential (48%) at 400 µg/mL while total antioxidant capacity of 59.8 µg AAE/mg was revealed at 400 µg/mL. No antiviral activity is reported on sabin like polio virus. Overall excellent biological properties are reported. We have shown that green synthesis can replace well established processes for synthesizing BiVO_4_ nanorods.

## Introduction

Phytosynthesis of nanoscaled materials is an innovative approach often considered as a potential replacement for various chemical or physical methods. The inherent nature of the chemical process often led to produce toxic wastes while the physical means are often accompanied with elevated energy requirements (Ovais et al. 2018b; Khalil et al. 2019a, b; Shah et al. 2018; Hassan et al. 2018). Relative to the biologically synthesized nanoparticles, the chemically synthesize nanoparticles indicate low biocompatibility and possess latent biological risks. In order to keep the energy balance and mitigating environmental risks, plants are used as a versatile bio-reductant for the synthesis of various metal nanoparticles or their nanocomposites. Medicinal plants possess a diverse reservoir of phytochemicals which can reduce and stabilize the nanoparticles (Ovais et al. 2018c; Mohamed et al. 2019). Metal and metal based nanomaterials have diverse applications in different fields and therefore a number of scientists have adopted novel methods for synthesis and application (Manikandan et al. 2017; Thema et al. 2016; Devika et al. 2012; Mwakikunga et al. 2010; Khamlich et al. 2011).

Metal vanadate have been frequently looked for potential applications as implantable cardiac defibrillators, batteries, catalysis and photo catalysis (Sivakumar et al. 2015). However, bismuth vanadate has emerged as a promising candidate due to its unique physiochemical, optical and ferro-elastic properties (Sarkar and Chattopadhyay 2012). Various applications of BiVO_4_ has been well studied in water splitting, sensors, pollutant degradation etc. (Ma et al. 2019; Vo et al. 2019; Prado et al. 2019; Jaihindh et al. 2019; Hassan et al. 2019; Chomkitichai et al. 2019). Recently, there has been growing interest in biological applications of BiVO_4_. The AgI–BiVO_4_ composite material indicated excellent potential for inactivation of *Escherichia coli* in water disinfection (Guan et al. 2018). Similarly, the octahedral shaped BiVO_4_ synthesized via hydrothermal approach revealed inactivation of *E. coli* (80% to 100%) (Sharma et al. 2016). 100% inactivation of *E. coli* after 30 min of exposure to Ag loaded BiVO_4_ is also reported (Regmi et al. 2018). BiVO_4_ is among few materials that remain stable in mild pH neutral conditions (Lichterman et al. 2013).

Due to the exciting properties and applications, there is considerable interest for the commercially scalable process for synthesizing BiVO_4_. Different methods like, ultrasonic-assisted, hydrothermal, pyrolysis, flame spray, chemical bath deposition, sonochemical, template-free solution and co-precipitation method have been explored to synthesize BiVO_4_ nanoparticles (Hu et al. 2018; Tao et al. 2019). Recently, plant extracts of *Callistemon viminalis* were used as a low cost reducing and stabilizing agents for biosynthesis of BiVO_4_ (Mohamed et al. 2018). Complementing to the limited literature on green avenues and biological properties of BiVO_4_, a green method was adopted by using dried fruit aqueous extracts of *Hyphaene thebaica* as green scaffolds for the synthesis of rod shaped BiVO_4_ which were subsequently studied for various biological properties. *H. thebaica* is a member of *Arecaceae*, locally referred as Doum (Arabic) and gingerbread tree (English). The medicinal applications of *H. thebaica* is well reported in the ethno-medicinal and folkloric scriptures (Khalil et al. 2019a, b). Various preparation of *H. thebaica* is reported for bleeding, haematuria, dyslipidemia, antihypertensive, diuretic diaphoretic, hypertension and lowering blood pressure etc. (Abdulazeez et al. 2019). In view of the medicinal applications and ethnopharmacological relevance, the fruit part of the *H. thebaica* was selected for biosynthesis.

## Materials and methods

### Processing of plants

The fruits of *H. thebaica* were obtained from (Aswan) Egypt, gently washed in running distill water for removing dust/impurities or any form of particulate matter, shade dried, powdered and used for extraction by heating 10 g of powdered fruit material to 400 mL of distil water at 100 °C/2 h on magnetic stirrer hotplate. Residual wastes were removed by filtering extracts for three times with Whattman filter paper and the remaining transparent extracts were used further.

### Biosynthesis of BiVO_4_

Bismuth nitrate (2.448 g) was added to 50 mL aqueous extracts and heated at 100 °C/1 h for ensuring complete dissolution of precursor salt. In a separate flask, VOSO_4_ (1.126 g) was introduced to 50 mL extracts and heated at 100 °C/1 h. Change in color was observed. Both solutions were mixed to make a mix of bismuth and vanadium ions, proportionally mixed to form bismuth vanadate. The resultant precipitates were washed three times by centrifugation and dried at 100 °C. The dried precipitate was annealed at 500 °C for 2 h in a tube furnace which yielded yellow colored powder assumed as BiVO_4_. Annealing was performed to obtain a high degree crystallinity and purity.

### Physical properties

Diverse techniques were applied to elucidate the main physical properties of green synthesized BiVO_4_. Powder X-ray diffraction was carried with diffractometer equipped with an irradiation line of 1.5406 A^0^ Cu Kα operating in Bragg–Brentano geometry. Debye Scherer formula was used to calculate the nano size while the data was compared with standard diffraction database. Vibrational characteristics were studied using Raman spectroscopy and FTIR. Diffuse reflectance spectra was recorded. Morphology was studied using HR-SEM and HR-TEM. Elemental composition was analyzed by Energy Dispersive Spectroscopy while Selected Area Electron Diffraction and zeta potential was also investigated. Once the physiochemical nature of the nanoparticles was established, they were then processed for analyzing their biomedical applications.

### Antimicrobial properties

Simple well diffusion assay as described earlier (Khalil et al. 2014) was used at different concentration to investigate the antibacterial and antifungal potential of the BiVO_4_ nanorods in the concentration range of 4 mg/mL to 250 µg/mL. Test bacterial strains were *Staphylococcus epidermidis* (ATCC 14490)*, Klebsiella pneumonia* (ATCC 13883)*, Bacillus subtilis* (ATCC 6633)*, Escherichia coli* (ATCC 15224) and *Pseudomonas aeruginosa* (ATCC 9721), while test fungal strains were *Aspergillus fumigates* (FCBP 66)*, Aspergillus flavus* (FCBP 0064)*, Aspergillus niger* (FCBP 0918)*, Mucor* sp. (FCBP 300) and *Fusarium solani* (FCBP 434). Briefly, the microbial cultures were standardized to an optical density of 0.5, corresponding to the MacFarland standards. 100 µL of inocula was dispensed on the Tryptic Soy Agar (bacterial media) and Sabouraud Dextrose Agar (fungal media) plates which was uniformly spread with sterile cotton swabs. Through sterile borer, 5 mm wells were made and 30 µL samples was introduced. Erythromycin and Amp B were used as positive control for bacteria and fungi respectively, while DMSO was added as a negative control. The bacterial cultures were incubated at 37 °C for 24 h while the fungal plates were incubated at 37 °C for 72 h. Zones of inhibition was measured and the MIC was considered as the least test concentration to cause microbial inhibition.

### Protein kinase inhibition

Streptomyces 85 E cultured on ISP4 medium was used to assess the PK inhibition as described previously (Fatima et al. 2015), from 4 mg/mL to 250 µg/mL. The standardized culture (100 µL) was dispensed on the media plates and spread uniformly. 5 mm borer was used to make wells and the test samples were introduced followed by incubation for 72 h at 30 °C. Bald and clear zones were measured while DMSO and Streptomycin were used as negative and positive controls respectively.

### Antioxidant assays

DPPH free radical scavenging and total antioxidant capacity were performed in the concentration range of 400–25 µg/mL, through a spectrophotometer based method as described previously (Hameed et al. 2019). The DPPH reagent solution was prepared by dissolving DPPH (9.6 mg) in methanol (100 mL). Test samples (20 µL) was added to DPPH reagent (180 µL), and the incubated for 20 min in dark. Results were recorded at 517 nm, and calculations were performed according to;

$$\% FRSA = \left[ {1 - {\raise0.7ex\hbox{${Ab_{S} }$} \!\mathord{\left/ {\vphantom {{Ab_{S} } {Ab_{C} }}}\right.\kern-0pt} \!\lower0.7ex\hbox{${Ab_{C} }$}}} \right] \times 100$$

Total antioxidant capacity (Karunakaran et al. 2016) was investigated using phosphomolybdenum based method and the results were expressed as ascorbic acid equivalents per milligram.

### Hemolysis

Hemolytic activity was performed as described previously (Malagoli 2007). Erythrocytes were isolated from freshly collected human blood in EDTA tubes, and their subsequent centrifugation at 14,000 RPM/5 min. 200 µL erythrocytes were added to 9.8 mL PBS for making erythrocytes suspension. The test nanoparticles in different concentrations were introduced in the Eppendorf tubes having an equal amount of the made erythrocytes suspension and incubated for 1 h at 35 °C. The reaction mix was then centrifuged at 10,000 RPM/10 min. Obtained supernatant was dispensed gently in 96 well plates and the hemoglobin release was monitored at 540 nm. Hemolysis was determined using the following formula;

$$\% Hemolysis = \left[ {\frac{{Ab_{S} - Ab_{NC} }}{{Ab_{PC} - Ab_{NC} }}} \right] \times 100$$

### Cell culture and antiviral experiments

Human Rhabdomyosarcoma Cells (RD), Human Laryngeal Carcinoma (HEp-2 cells) and L20B cells (mouse fibroblast cells) were enriched in Eagle’s Minimal Essential Medium (E’MEM) containing (10%) FBS. Propagation of Sabin like Poliovirus (Type 1) was done through HEp-2 cells supplemented with 2% FBS. Viral titers were determined using Karber formula after titration of the virus on RD cell (Thuy et al. 2013).

### Assessment of cytotoxicity

MTT assay was used for cytotoxicity assessment with slight modifications (Lin et al. 2005). MTT assay is based on the mitochondrial dehydrogenase of viable cells, giving blue formazan product quantified spectrophotometrically. MTT assay was performed in 96-well plates, seeded with 100 µL RD cells, HEp-2 cells and L20B cells at a concentration of 3.5 × 10^5^ cells/mL cultured in E’MEM (200 μL) containing FBS (10%) and incubated at 36 °C for 48 h in CO_2_ incubator to maintain a stable normal cell monolayers. Afterwards cells were treated with different doses of BiVO_4_ NPs (1000–15 μg/mL), and incubated for an additional 48 h at 36 °C. Cells were examined daily under inverted light microscope to determine the minimum concentration of BiVO_4_ NPs resulting in morphological changes in cells. 100 μL of MTT solution (5 mg/mL) was introduced to wells after removing the media and incubated (4 h/37 °C). MTT solution was then discarded and 50 μL dimethyl sulfoxide (DMSO) was added to dissolve insoluble formazan crystals and incubated (37 °C/30 min). Optical density (OD) was measured at 540 nm using a spectrophotometer reader (victor × 3, Perkin Elmer). Data were obtained from triplicate wells. Cell viability was expressed with respect to the absorbance of the control wells (untreated cells), which were considered as 100% of absorbance. The percentage of cytotoxicity is calculated as

$$\% viability = \frac{A - B}{A} \times 100$$

where A and B are the OD540 of untreated and of treated cells, respectively. The 50% cytotoxic concentration (CC50) was defined as the compound’s concentration (μg/mL) required for the reduction of cell viability by 50%.

### Assessment of antiviral activity

Confluent RD, Hep2C and L20B cell culture were treated with mixture of BiVO_4_ NPs and virus dilutions. Firstly, 100TCID_50_ poliovirus type 1 were diluted tenfold into two concentration of 10TCID_50_ and 1TCID_50_ in 2% E’MEM and introduce to non-cytotoxic concentrations of BiVO_4_ NPs (15 µg/mL) in ratio of 1:1 (v/v) and incubated for 1 h at 36 ^°^C. After that, mixture of virus dilutions (100TCID, 10TCID50 and 1TCID_50_) was incubated with BiVO_4_ NPs (1 mg/mL to 15 µg/mL) in 96 well plate seeded with healthy monolayer of Hep2C cells (3.5 × 10^5^ cells/mL) in a CO_2_ incubator with 5% CO_2_. Cell growth 10% EMEM medium was decanted and replaced with 200 µL media respectively. Three controls were used including: (i) 50 μL of BiVO_4_ NPs at 15 µg/mL concentration (without poliovirus) were added to wells containing RD cells for BiVO_4_ NPs control (Magudieshwaran et al. 2019); 50 µL of poliovirus at concentration of 1TCID_50_, 10TCID_50_ and 100TCID_50_ was added to wells (iii) 200 μL of fresh maintenance medium was added for negative controls. The cultures were incubated at 36 °C post-infection, and cytopathic effect (CPE) was daily observed by inverted light microscopy. The cellular viability was determined through staining method using crystal violet. Optical density (OD) was measured at 490 nm using a spectrophotometer.

## Results

### Physical characterizations

**Fig. 1:**
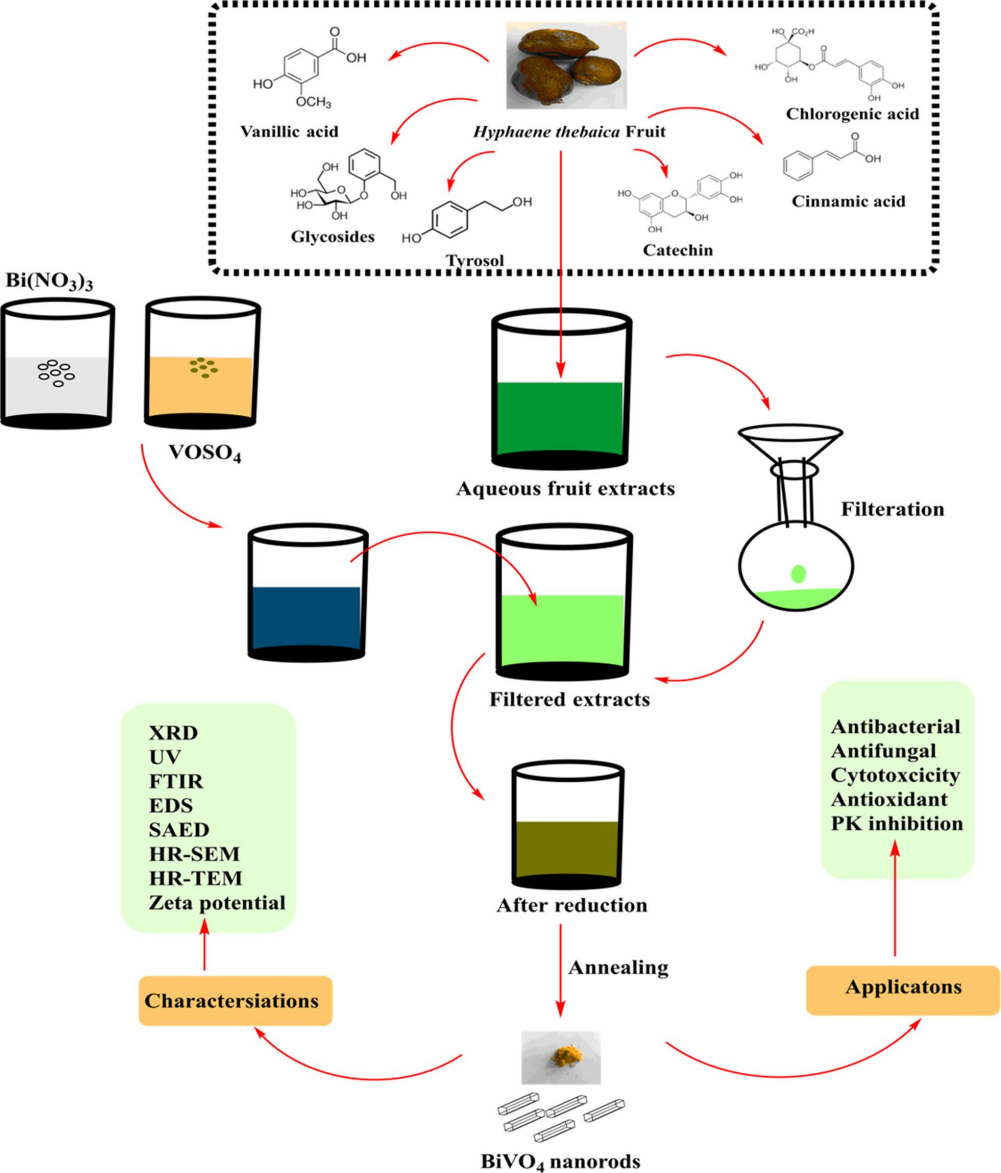
A schematic representation of the design of study

**Fig. 2:**
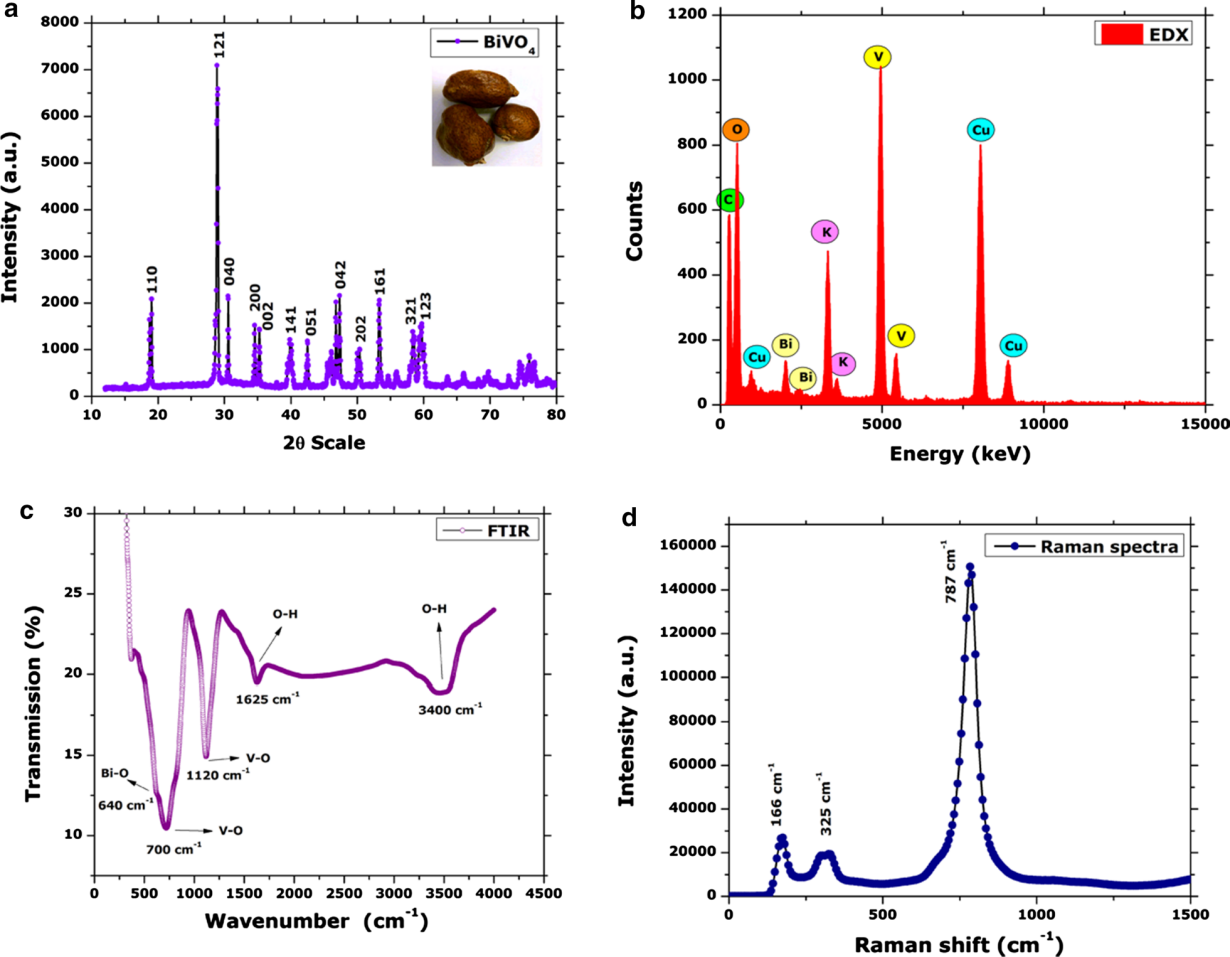
Various spectroscopic characterisation performed on BiVO_4_. **a** X-ray diffraction pattern; **b** Energy Dispersive Spectra; **c** Fourier Transformed Infrared Spectra; **d** Raman Spectra

*Hyphaene thebaica* dried fruit aqueous extracts were used as bio reductant for synthesis of novel BiVO_4_ nanorods. Different techniques were used to characterize the room temperature physiochemical properties of the nanorods. The overall process and study scheme has been summarized in Fig. 1. The extracts were treated separately with precursor salts of bismuth and vanadium giving light brown and blue color. Powdered X-ray diffraction was carried out to reveal the crystallographic properties and presence of BiVO_4_ nanorods. Figure 2a indicate the XRD spectra. Bragg peaks are observed at 18.6°, 28.9°, 30.5°, 34.4°, 35.2°, 39.5°, 42.4° 47.3°, 50.3°, 53.3°, 58.5° and 59.2° on 2 theta scale that corresponds to the crystallographic reflections of (110), (121), (040), (200), (002), (141), (051), (042), (202), (161), (321) and (123) respectively. These crystallographic peaks were in correspondence with the JCPDS pattern 00-014-0688 for Clinobisvanite phase monoclinic bismuth vanadium oxide (BiVO_4_). Sharpness of the peaks indicate a highly crystalline nature of the BiVO_4_. No other peaks were detected which suggested the single phase purity of the BiVO_4_ nanorods. The crystal structure belonged to space group I2/a with lattice parameters were deduced as 〈a〉 = 5.1 A°, 〈b〉 = 11.7 A°, and 〈c〉 = 5.09 A° correlating to the BiVO_4_ with yellow color. Scherer approximation revealed average size of ~ 7 nm as indicated in Table 1A.

**Table 1:** A: Major values deduced from XRD data of BiVO_4_ nanoparticles from *H. thebaica*; B: Zeta potential report of BiVO_4_ nanorods

(A) XRD calculations					
(hkl)	2Ɵ	Ɵ	Ɵ (rad)	FWHM	Average size (nm)
110	18.62	9.31	0.162556	0.12	11.77878
121	18.92	9.46	0.165175	0.12	11.78388
040	28.88	14.44	0.252127	0.25	5.761433
200	30.49	15.245	0.266183	0.16	9.035849
002	34.45	17.225	0.300754	0.17	8.590418
141	35.15	17.575	0.306865	0.18	8.128732
051	39.72	19.86	0.346762	0.81	1.830984
042	45.99	22.995	0.4015	0.11	13.77568
202	46.67	23.335	0.407437	0.24	6.329919
161	47.23	23.615	0.412325	0.22	6.920051
321	50.25	25.125	0.43869	0.65	2.370326
123	53.18	26.59	0.46427	0.36	4.333172
Average grain size (nm)					7.543515

(B) Zeta potential measurements	
Zeta potential (mV)	− 5.21
Zeta deviation (mV)	5.94
Conductivity (mS/cm)	0.0217

After establishing single phase purity of BiVO_4_ nanorods, their elemental analysis were carried out using Energy Dispersive Spectroscopy as indicated in Fig. 2b. Spectral analysis confirmed the presence of “Bi”, “V” and “O”. The peak of “C” relates to the grid support. Some traces of “Cu” and “K” were also found that most probably emanates from the organic components of the fruit material.

Figure 2c indicate the FTIR spectra of the biosynthesized BiVO_4_ nanorods from 200 to 4000 cm^−1^. Main absorption peaks were observed centered at ~ 640 cm^−1^, ~ 700 cm^−1^, ~ 1120 cm^−1^, ~ 1625 cm^−1^ and 3400 cm^−1^. Peak centered at ~ 640 cm^−1^ can be ascribed to Bi–O (bending) while at ~ 700 cm^−1^ and ~ 1120 cm^−1^ to V–O symmetric and asymmetric vibrations (Khan et al. 2017). IR peaks centered at ~ 1625 cm^−1^ and 3400 cm^−1^ can be ascribed to the stretching vibrations of O–H group.

**Fig. 3:**
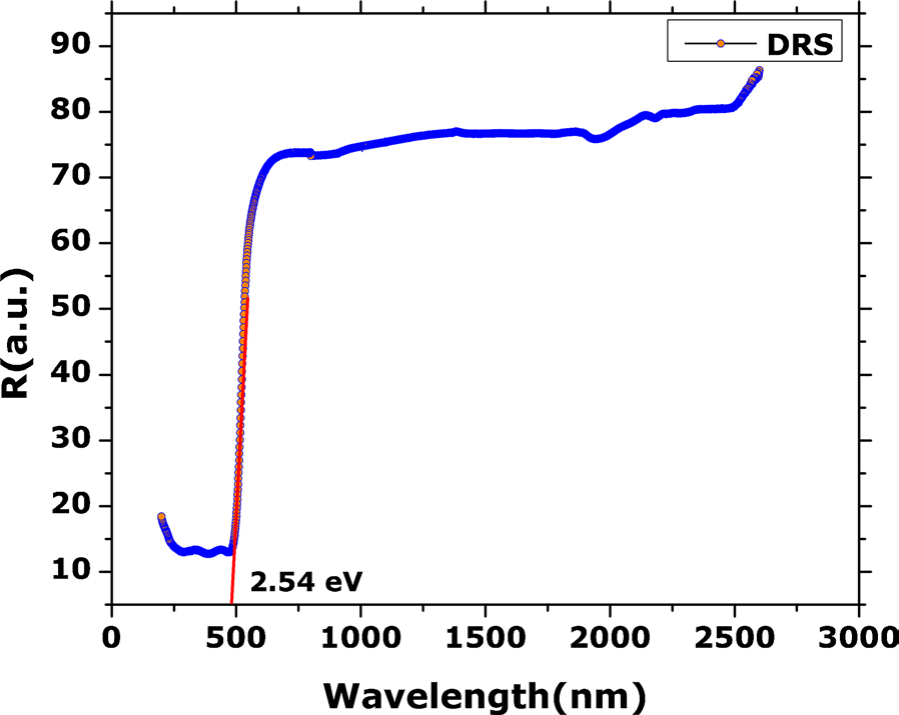
Diffuse reflectance spectra of BiVO_4_ nanorods

Raman spectroscopy is considered as a powerful technique to probe structure of metal oxides. Raman spectroscopy was carried out to further elaborate the vibrational properties of BiVO_4_ nanorods in the spectral range of 0 cm^−1^ to 1500 cm^−1^. Three noticeable raman peaks were observed centered at 166 cm^−1^, 325 cm^−1^ and 787 cm^−1^. The intense stretching mode of VO_4_ is observed at 787 cm^−1^. Raman peak centered at 325 cm^−1^ represents the asymmetric bending mode of VO_4_ tetrahedron (Brack et al. 2015). Peak centered at 166 cm^−1^, is attributed to the external mode vibration (Xu et al. 2018; Nikam and Joshi 2016). The raman spectra of BiVO_4_ nanorods is indicated in Fig. 2d. The UV–Vis diffuse reflectance spectrum was recorded from 0 to 3000 nm. The BiVO_4_ nanorods revealed good visible light absorption with absorption edge at 487 nm. The steep shape is ascribed to the band gap transitions. The energy of the band gap is estimated to be ~ 2.54 eV. DRS spectra has been indicated in Fig. 3.

**Fig. 4:**
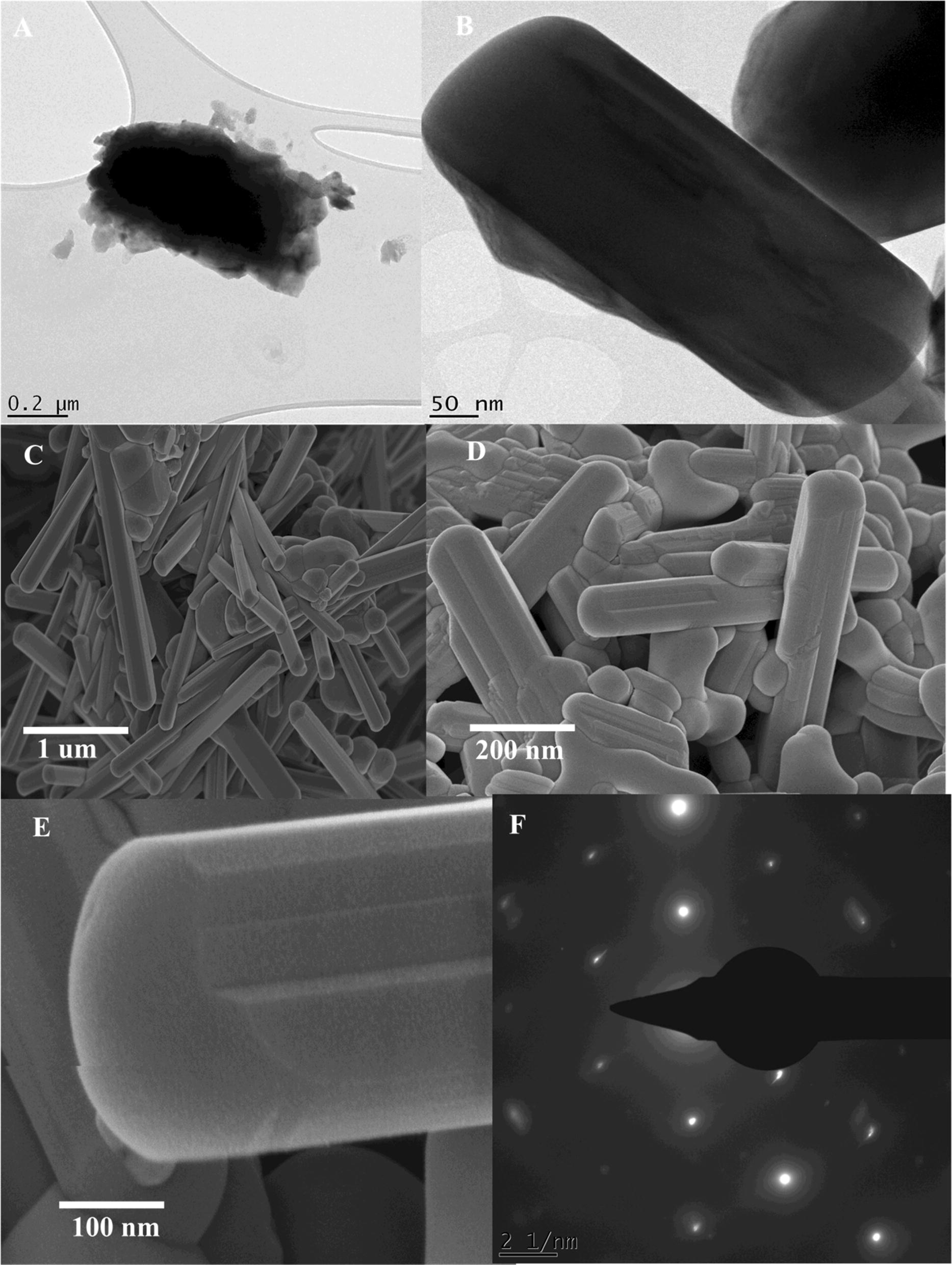
High resolution microscopic images of BiVO_4_ nanorods; **A**, **B** HR-TEM images; **C**–**E** HR-SEM images; **F** SAED pattern

Inset Fig. 4A–F indicate the various high resolution microscopic images of the synthesized nanoparticles to establish their morphology. One can conclude the formation of well aligned rod shape of the BiVO_4_. The Selected Area Electron Diffraction pattern suggest crystalline nature of the nanorods as indicated in Fig. 4F. Zeta potential of the BiVO_4_ nanorods was recorded as − 5.21 mV. Results are indicated in Table 1B.

### Antimicrobial properties

**Fig. 5:**
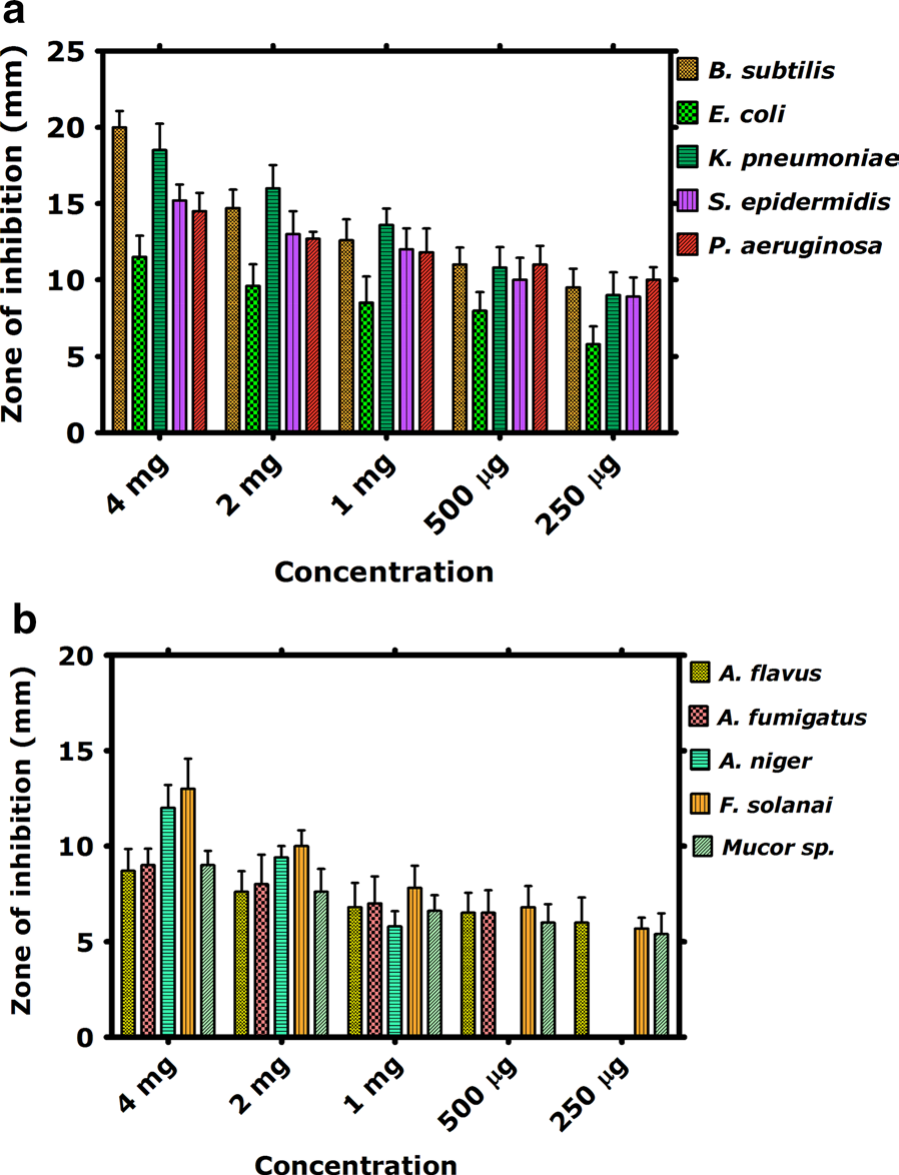
Antibacterial and antifungal potential of BiVO_4_ nanorods across different concentrations. **a** Antibacterial activities; **b** antifungal activities

The antimicrobial properties of the BiVO_4_ nanorods have been explored against various bacterial and fungal strains. Results of the antibacterial and antifungal properties are indicated in Fig. 5a, b. Among the tested bacterial strains, *B. subtilis* revealed highest zone of inhibition (20 mm to 9.5 mm) in the concentration range of 4 mg/mL to 250 µg/mL. The least susceptible strain was found to be *E. coli* which revealed maximum zone of inhibition (11.5 mm) at 4 mg/mL. The order of the antibacterial activity of BiVO_4_ nanorods was found as *B. subtilis* > *K. pneumoniae* > *S. epidermidis* > *P. aeruginosa* > *E. coli.* Interestingly for *P. aeruginosa* and *S. epidermidis,* the observed zone of inhibition was much larger than the positive control Erythromycin at the rate of 1 mg/mL. Similarly, against *K. pneumoniae,* the BiVO_4_ nanorods were found to be as effective as the positive control.

**Fig. 6:**
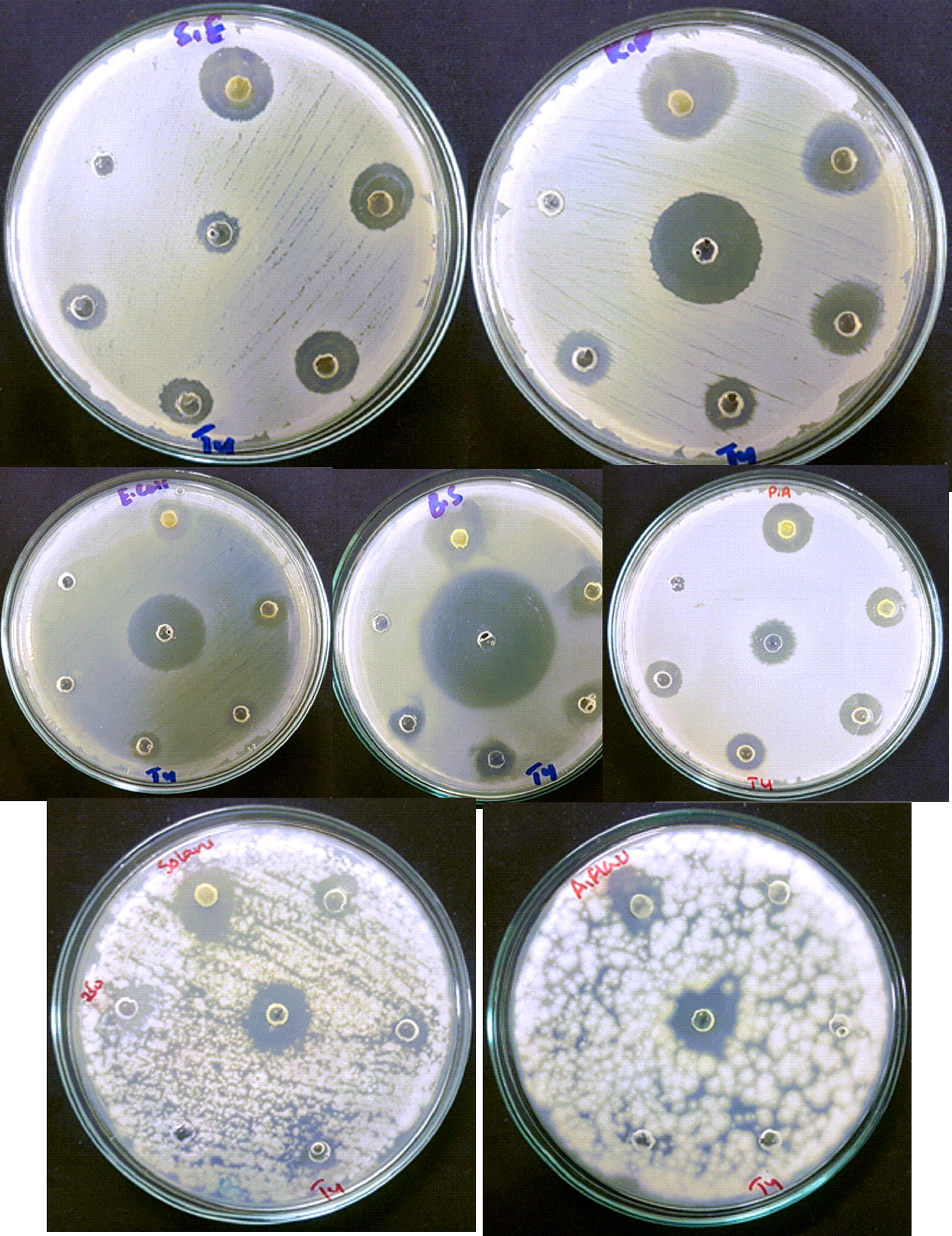
Selected images of the antimicrobial assays

Among the five tested fungal strains, *F. solani* was observed as most susceptible fungal strain revealing zones ranging from 13 to 5.7 mm at the tested concentrations of BiVO_4_ nanorods. The order by which the antifungal potential observed was *F. solani *> *A. niger* > *Mucor* sp. > *A. fumigatus* > *A. flavus. A. niger* did not revealed any zones at 500 µg/mL or below, while *A. fumigatus* was found to be in effective at 250 µg/mL. Against *F. solani* and *A. niger*, the zones were revealed similar to the zones obtained from positive control (Amp B). Results of antifungal activity are summarized in Fig. 5b. Figure 6 indicate various selected images of the antibacterial and antifungal activities. Moreover, these activities revealed a dose dependent response.

### Protein kinase inhibition

**Fig. 7:**
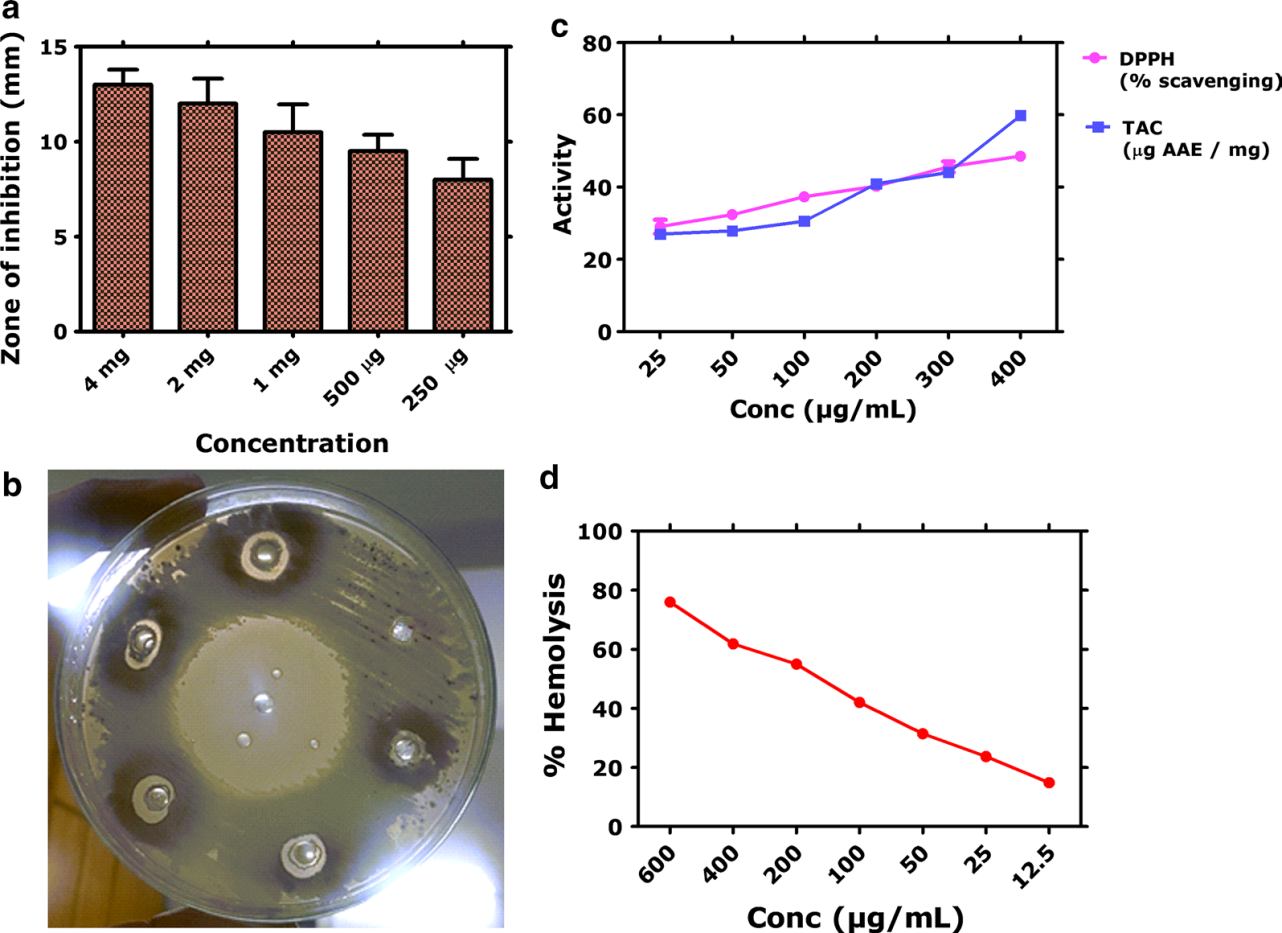
PK, antioxidant and hemolytic potential. **a**, **b** Protein kinase inhibition; **c** Antioxidant potential; **d** Hemolytic potential

A simple assay based on the Streptomyces 85 E strain is used to screen PK inhibitors. Figure 7a, b indicate the protein kinase inhibition potential of the *H. thebaica* mediated BiVO_4_ nanorods. Excellent PK inhibition was revealed. The zones of inhibition at the tested concentration ranged from 13 to 8 mm. However, the zones of inhibition was much smaller then obtained for positive control.

### Antioxidant assays

The antioxidant potential of the BiVO_4_ nanorods was determined using DPPH free radical scavenging and total antioxidant capacity. Moderate free radical scavenging potential is reported. At the highest tested concentration of 400 µg/mL, the percent scavenging was found to be 48%, which gradually declined as the concentration was lowered below 400 µg/mL. At the lowest tested concentration (25 µg/mL) of BiVO_4_ nanorods, 29% scavenging was observed. These results were complemented by the total antioxidant capacity which was determined as µg AAE/mg. Highest value (59.8 µg AAE/mg) of the ascorbic acid equivalents (AAE) was reported at 400 µg/mL while at lowest concentrations of 25 µg/mL, 26.9 µg AAE/mg was reported. Overall the antioxidant activity can be concluded as moderate and dose dependent. Results of antioxidant potential is indicated in Fig. 7c.

### Hemolysis

Erythrocytes lysis assay was performed to evaluate the toxicity of BiVO_4_ on fresh isolated RBCs in test concentrations ranging from 600 to 12.5 µg/mL. The BiVO_4_ nanorods were observed to cause increased degree of hemolysis (75%) at higher concentrations 600 µg/mL, while percent hemolytic potential decreased with decrease in concentration. At lowest tested concentration (12.5 µg/mL), 14.8% hemolysis was observed. Overall, significant hemolytic nature of the BiVO_4_ nanorods is observed. Results are indicated in Fig. 7d.

### Cytotoxicity of BiVO_4_ (RD cells, Hep2C and L20B)

**Fig. 8:**
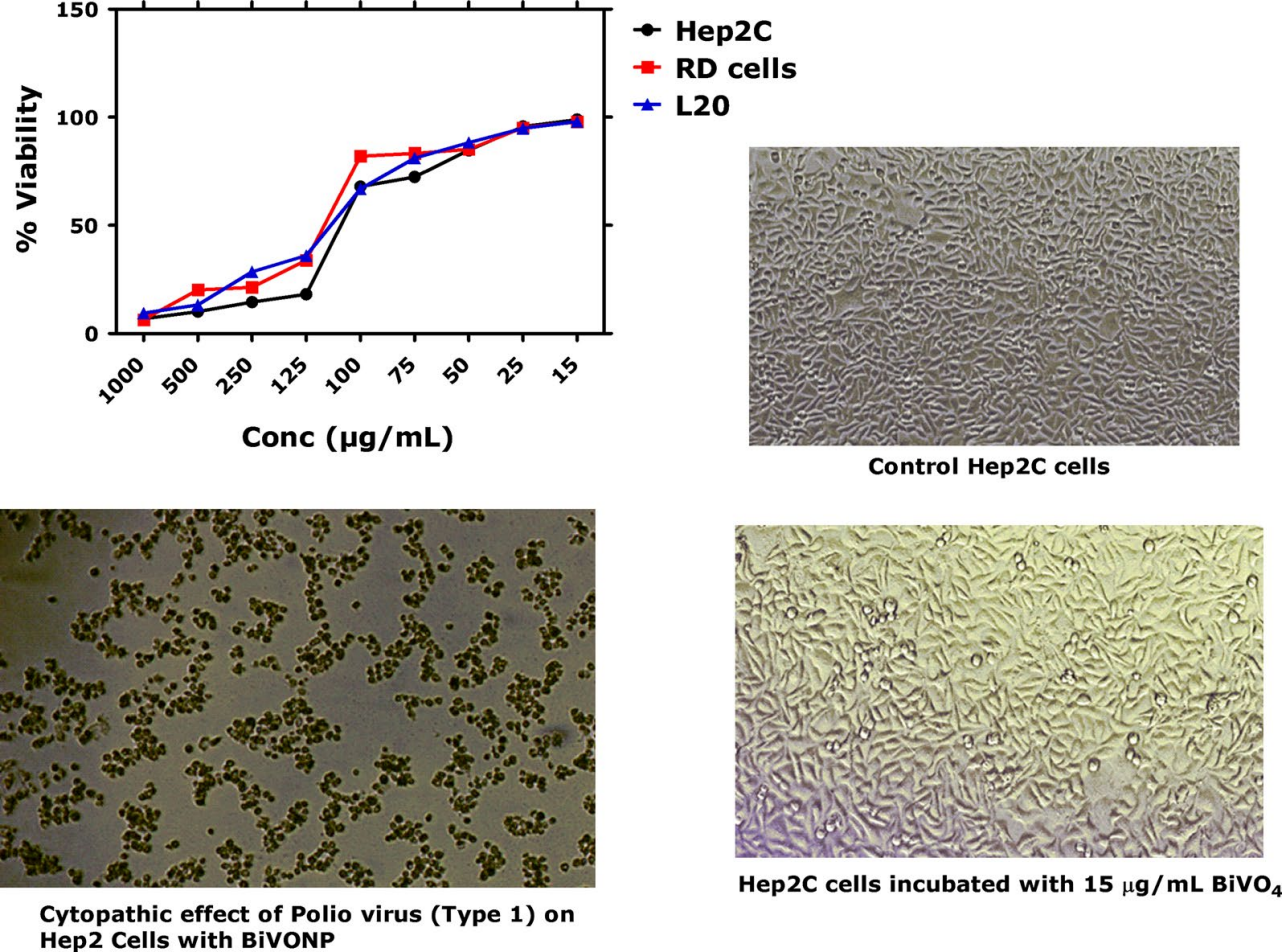
Cytotoxicity of BiVO_4_ nanorods on different cells and investigating their anti-polio virus potential

Figure 8 indicate the experimental results revealed ~ 95% cell viability of RD cells (Human Rhabdomyosarcoma cells), Hep2C cells (Human Laryngeal Carcinoma) and L20B cells (Mouse Fibroblast cells) after incubation with both BiVO_4_ NPs (15 µg/mL). At higher concentration of BiVO_4_ NPs, the viability of cells started decreasing slightly compared to negative control. No cytopathic effects was indicated RD, Hep2C and L20 cells was revealed indicating compatibility of cell cultures with biological synthesized BiVO_4_ NPs after 2 h and 48 h incubation time. These obtained results suggest that both synthesized BiVO_4_ NPs are nontoxic to cells up to 48 h post-incubation at low concentration.

### Antiviral activity of BiVO_4_

In order to investigate the antiviral activity of BiVO_4_, three concentrations (1TCID_50_, 10TCID_50_ and 100TCID_50_) of Sabin like poliovirus (Type 1) were incubated with BiVO_4_ NPs (15 µg/mL). Our results indicated that cells remained viable at 24 h post-infection. At 5th day of incubation, it was observed that most Hep2C cells were destroyed at viral concentration of 100TCID50, 10TCID_50_ and 1TCID_50_ across all the tested concentrations of BiVO_4_ NPs. It can be inferred that the BiVO_4_ nanorods were unable to inhibit the propagation of polio virus in the Hep2C cells. Complete destruction of the Hep2C cells was due to the intracellular propagation of the polio virus in Hep2C, cultured with 15 µg/mL of the BiVO_4_ nanorods.

## Discussion

The interface of green nanotechnologies and medicinal plants have delivered excellent results over the previous decades. A number of biogenic metal based nanoparticles has revealed excellent results (Sathiyavimal et al. 2018). Green synthesized nanoparticles often exhibit multifunctional nature and therefore can be applied in diverse applications (Nasar et al. 2019; Venugopal et al. 2017). The interesting properties and potential applications of BiVO_4_ has fueled the growing research on their synthesis procedures which easy, scalable, green and cost effective. Different chemical and physical processes have been adopted for the synthesis of BiVO_4_, however, the potential of biological resources in their synthesis is largely untapped. Recently, we have established the successful synthesis of BiVO_4_ by using *Callistemon viminalis* floral extracts as bioreductant (Mohamed et al. 2018). Herein, a further detailed study was conducted on the physical as well as biological properties of BiVO_4_ nanorods, synthesized using the fruit extracts of *H. thebaica*. Plant extracts are reported to have a rich chemistry which has the tendency to catalyze redox reactions and subsequently stabilize the nanoparticles. The phytochemicals that usually take part in the reduction are mostly considered to be phenols, flavonoids, citric acid, membrane proteins, reductases, dehydrogenases etc. while the stabilizing moieties can be tannic acids, extracellular proteins, peptides, enzymes (Karatoprak et al. 2017; Akhtar et al. 2013; Elegbede et al. 2018). *H. thebaica* extracts are rich in the phenolic like cinnamic acid, sinapic acid, chlorogenic acid, vanillic acid, Epicatechin, caffeic acid, coumarin and flavonoids like quercetin, hesperetin, naringin, glycosides, rutin (El-Beltagi et al. 2018) etc. which can serve as bioreductant and capping agents in biosynthesis of BiVO_4_ nanorods. This method is easily scalable, environmentally benign and easy to manage. Physical characterisation techniques established the unique structural and morphological nature of BiVO_4_. XRD data revealed Clinobisvanite phase of BiVO_4_ and the obtained peaks are consistent with previous results (Gawande and Thakare 2012; Sivakumar et al. 2015). In literature, three polymorphs of BiVO_4_ are reported which are Pucherite (orthorhombic), Dreyerite (tetragonal) and Clinobisvanite (monoclinic). Among these mineral forms, Clinobisvanite is most stable thermodynamically and possess significant photocatalytic potential (Zhao et al. 2011). Depending on the conditions, ferroelastic monoclonal-tetragonal-phase transitions are reported (Frost et al. 2006). The elemental analysis confirms the presence of “Bi”, “V” and “O” which establishes the synthesis of BiVO_4_. The infrared spectra of the synthesized nanorods affirms the potential role of phenolic components in plant extracts that have catalyzed the reduction and stabilization of BiVO_4_ nanorods. The role of phenolic compounds as reducing agents is well established (Ovais et al. 2018a; Soto et al. 2019). The role of Sulphur and Nitrogen rich protein compounds are also considered to play a significant role (Ballottin et al. 2016). The IR peaks obtained for Bi–O bending vibrations and V–O symmetric and asymmetric vibrations are consistent with previous studies (Khan et al. 2017). The Raman peaks were found to be in agreement with reported studies (Nikam and Joshi 2016; Xu et al. 2018). The shape and morphology of the BiVO_4_ was investigated as to be nanorods. The nanorods shaped morphology of the BiVO_4_ has been reported in the literature (Dubal et al. 2018; Liu et al. 2019; Chen and Lin 2018). Zeta potential revealed a value of − 5.21 mV. The presence of negative charge suggest the presence of electrostatic repulsive forces which intends to repel the particles from one another, therefore, enhances stability by preventing aggregation. The surface charge on the nanoparticles and local environment plays an important role in determination of zeta potential (Chaudhuri and Malodia 2017).

To date, most of the studies revealing the antimicrobial potential of BiVO_4_ considered only the waste water disinfection. Recently, an innovative photocatalytic fabricated by Ni doping on BiVO_4_ revealed excellent degradation of ibuprofen (80%) within 90 min, while 92% reduction of *E. coli* after 5 h exposure to light was recorded. In addition, Ni-BiVO_4_ indicated excellent anti algal potential (Regmi et al. 2017). A novel BiVO_4_/InVO_4_ nanocomposite material revealed excellent sterilization potential against various bacterial strains i.e*. E. coli* (99.71%), *S. aureus* (99.55%), *P. aeruginosa* (99.54%) and *A. carterae* (96%) (Zhang et al. 2019). In a recent report, graphene based nanocomposite of BiVO_4_ (90 mg/L) was studied for antibacterial potential against *B. subtilis* and *S. aureus* using disc diffusion assay with, but no zone of inhibition was observed, suggesting a nontoxic nature of the BiVO_4_–GO nanocomposite (Zhao et al. 2019). Our work describe for the first time the antimicrobial potential of the phytosynthesized BiVO_4_. The physiochemical nature of the nanorods (surface coating, reducing-stabilizing agents, shape, size, surface morphology) plays important role in determining the antimicrobial activities (Zhang et al. 2016). The mechanism that drives the antimicrobial potential of the metal nanoparticles has mostly been attributed to the generation of reactive oxygen species. The present age of antibiotic resistance signifies the need to develop alternative antibiotics. The microorganisms tends to smartly evolve in order to develop resistance to the available treatments at a speedy rate. Furthermore, new antibiotics are not produced at the same pace at which microorganisms are getting resistant. Novel approaches like nanoantibiotics are considered vital to curb antibiotic resistance. BiVO_4_ nanorods have indicated excellent antimicrobial activities and therefore can be considered as a novel nanoantibiotics for future, before detailed evaluation of toxicity. The inhibition of protein kinase enzymes is considered a popular target for the anticancer therapies. Therefore, tremendous research has been devoted for identifying potent inhibitors of PK enzymes. Protein kinase are responsible for phosphorylating serine-threonine and tyrosine amino acids and play integral role in signaling differentiation and division of cells. The malfunctioned phosphorylation leads to the progression of cancer. By inhibiting the protein kinase that serve as a bridge for the signaling factors, cell division can be stopped ultimately hindering cancer progression. PK enzymes are vital for the growth of hyphae in Streptomyces 85 E strain and therefore, considered as a model organism. The cell culture experiments suggested the viability of the cells at low concentrations of the BiVO_4_ nanorods.

With the advances of the metal nanoparticles research medicinal plants have emerged as an exciting resource to be explored from green synthesis aspects. We have reported the biosynthesis of BiVO_4_ nanorods using *H. thebaica* fruit extracts as a low cost and green templating agents and studied them for possible biological applications. Excellent antibacterial and antifungal activities are reported. BiVO_4_ nanorods were most effective on *Bacillus subtilis* and *Fusarium solani.* Good protein kinase inhibition and antioxidant potential is revealed. The BiVO_4_ induced hemolysis at high concentrations. At low concentrations, the cell culture experiments revealed compatibility and non-toxicity. No potential antiviral activity was identified for BiVO_4_.

Green synthesis using medicinal plant extracts provides an excellent platform for assembling nanomaterials for different applications. The process is not only economical but converging evidence suggests enhanced compatibility of the biosynthesized nanoparticles making them ideal for nanomedicinal applications. Most of the work in this area has been dedicated to the silver and gold nanoparticles and their nanomedicinal applications which necessitates the need of extending this methodology to novel nanomaterials. BiVO_4_ has diverse applications in industries and further research is encouraged to use to different plant extracts to synthesize BiVO_4_ and explore their biomedical potential.

## Data Availability

All data material is available for use.
